# Mechanical stimulation prevents impairment of axon growth and overcompensates microtubule destabilization in cellular models of Alzheimer’s disease and related Tau pathologies

**DOI:** 10.3389/fmed.2025.1519628

**Published:** 2025-05-14

**Authors:** Alice Alessandra Galeotti, Lorenzo Santucci, Jennifer Klimek, Mohamed Aghyad Al Kabbani, Hans Zempel, Vittoria Raffa

**Affiliations:** ^1^Department of Biology, University of Pisa, Pisa, Italy; ^2^Faculty of Medicine and University Hospital Cologne, Institute of Human Genetics, University of Cologne, Cologne, Germany; ^3^Center for Molecular Medicine Cologne (CMMC), University of Cologne, Cologne, Germany

**Keywords:** nano-pulling, mechanical stimulation, microtubule stabilization, Alzheimer’s disease, Tau pathology

## Abstract

Alzheimer’s disease (AD) and related tauopathies such as frontotemporal dementia (FTD) or traumatic brain injury (TBI) are neurodegenerative disorders characterized by progressive loss of memory and cognitive function. The main histopathological features of AD are amyloid-*β* plaques and Tau neurofibrillary tangles, suggested to interfere with neuronal function and to cause microtubule (MT) destabilization. We recently demonstrated that low mechanical forces promote MT stabilization, which in turn promotes axon growth and neuronal maturation. As neurites may become dystrophic due to MT destabilization in tauopathies, we hypothesized that force-induced MT stabilization is neuroprotective in cell models subjected to tauopathy-like stress. We set up two different pathological cellular models subjecting them to AD-related Tau pathology stressors. We found that exposure of mouse primary neurons to Tau oligomers and neurons derived from human induced pluripotent stem cell (hiPSC) to amyloid-*β* oligomers resulted in neurotoxic effects such as axonal shortening, reduction in dendrite number, and MT destabilization. Mechanical stimulation (i) prevented delays in axonal extensions and dendrite sprouting, restoring axon outgrowth to physiological levels, and (ii) compensated for axonal MT destabilization by increasing MT stability to levels higher than in control conditions. In summary, we here demonstrate that low mechanical force can be used as a neuroprotective extrinsic factor to prevent MT destabilization and axon degeneration caused by AD-like or tauopathy-like stressors.

## 1 Introduction

Alzheimer’s disease (AD) and related tauopathies such as frontotemporal dementia (FTD) or traumatic brain injury (TBI) are neurodegenerative disorders characterized by progressive loss of memory and cognitive function. While this is in the late stages accompanied by overt cell loss, earlier stages in AD/tauopathy are associated with more subtle effects such as synapse, neurite, and cytoskeleton dysfunction. The main histopathological features of AD are extracellular amyloid-*β* (Aβ) plaques and intracellular microtubule-associated protein (MAP) Tau neurofibrillary tangles (NFT) (1). Aβ plaques are aggregates made up of incorrectly folded peptides that derive from aberrant cleavage of the transmembrane Amyloid Precursor Protein (APP), a cell surface receptor with physiological functions relevant to neurite growth, neuronal adhesion, axonogenesis, and synaptogenesis (2). Tau pathology is the hallmark of several neurodegenerative disorders belonging to the category of tauopathies, of which AD is the most common. In AD, according to the amyloid cascade hypothesis, the presence of Aβ aggregates triggers the changes that lead to Tau pathogenic cascade resulting in Tau missorting and aggregation, neuronal dysfunction, neurite retraction, and eventually cell death (2, 3). Despite recent genetic evidence demonstrated the existence of a protective APP variant (4, 5) and partially effective anti-amyloid treatments are emerging (6, 7), the amyloid cascade hypothesis is disputed: Braak and Braak, renowned for their staging of AD based on Tau pathology, have presented findings that challenge the centrality of the amyloid cascade hypothesis. Their work demonstrated that Tau pathology often appears first in the entorhinal cortex, frequently without the presence of senile plaques (SPs), which are associated with (Aβ) deposition (8, 9). This observation suggests that tau-related NFT may precede amyloid deposition in the pathological sequence of AD. In the overwhelming majority of tauopathies, the initiator of disease is anyway not amyloid-*β*, but either physical trauma resulting most likely in axonal disruption due to shearing forces such as in TBI (10), a range of genetic disorders leading to Tau pathology as a secondary event, or Tau itself [e.g., Tau pathogenic mutations resulting in a subset of FTD (11). Tau protein has the physiological functions of binding microtubules (MTs), controlling their assembly and stabilizing them, therefore regulating axonal transport, neurons growth, and synapse establishment (12). Alterations in post-translational modifications, including hyperphosphorylation, cause a conformational change in Tau that underlies the pathogenesis of AD and related tauopathies, leading to the formation of intracellular NFT (13). Tau dissociates from MTs and is sequestered into these insoluble aggregates, causing MT destabilization and loss from axons. These pathological conditions disrupt axonal transport and synapses and ultimately lead to neuronal death and loss of cognitive function (14–16). Since MTs play a key role in neuron function, being involved in axonal transport, research in the area of new therapeutic strategies for AD and related tauopathies included also MT-stabilizing agents in recent years (17, 18). Recently, mechanical force has emerged as an extrinsic factor that stabilizes MTs (19). It has been suggested that MTs are inherently mechano-sensitive, and computational studies indicate that pulling forces (in the range of a few piconewtons) stimulate MT assembly by promoting the formation of lateral non-covalent bonds between GTP-tubulin dimers, thereby aiding the closure of the protofilament wall (20). Supporting this prediction, increased traction forces have been shown to slow down MT depolymerization (21). Evidence supporting the role of MTs as mechanosensors comes from classical micromanipulation experiments, which showed that traction forces ranging from 0.5 to 2 piconewtons (pN) decrease the likelihood of growing MTs of undergoing shrinkage, while increasing the probability of short MTs of resuming growth (22). Moreover, force can indirectly affect MT stability by modulating the activity of MAPs. For instance, studies on interactions between MTs and molecular motors have already shown that force influences the probability of MT/MAP association (23, 24). Similarly, it has been found that force can modulate interactions between MTs and other MAPs, such as proteins that associate with the plus end. XMAP215, an enzyme that catalyzes MT growth by adding tubulin dimers to the MT plus end, was found to increase the rate of MT growth under a 1 pN force application (25), possibly by enhancing enzyme activity. Interestingly, compressive forces also seem to affect MTs by causing the re-localization of plus-tip proteins [EB1 and CLASP2] from the microtubule end to the microtubule shaft (26). Recently, our group set up a method named nano-pulling (based on the use of magnetic nanoparticles (MNPs) in presence of magnetic fields) to mechanically stimulate neurons *in vitro* chronically by generation of a pulling force in the order of 10 pN (27–33). We used nano-pulling to test the effect of extremely low forces applied chronically in neural cells. We found, in both mature neurons (hippocampal neurons and dorsal root ganglion neurons) and neural progenitor cells (NPCs) undergoing neural differentiation, that force induces axonal MT stabilization (29, 32–34). When axonal MTs become more stable, their turnover rate declines, leading to their accumulation. Since MTs serve as the primary cytoskeletal “tracks” for axonal transport, this accumulation results in a local build-up of vesicles and organelles within the axon, which are components of translation platforms. This, in turn, raises the likelihood of forming translational platforms (35). Such positive regulation of axonal transport and local translation helps facilitate the addition of new mass required for axon growth (19, 32).

Since MT destabilization is one of the key features of AD and suggested for other tauopathies (36, 37), findings regarding MT stabilization in response to force could be very relevant to tauopathies and possibly for AD. In this study, we explored the hypothesis that mechanical stimulation of neurons could be exploited as a novel neuroprotective strategy to prevent or decrease MT instability and its neurotoxic effects in AD and related tauopathies. The present study represents a proof of concept, aimed at investigating the neuroprotective effect of mechanical force in *in vitro* models. We set up two different pathological cellular models using mature and immature neurons and subjecting them to AD-related Tau pathology stressors. Specifically, we treated primary mouse hippocampal neurons (HNs) with Tau protein oligomers (oTau), and NPCs derived from human induced pluripotent stem cells (hiPSCs) undergoing neuronal differentiation in cortical neurons with amyloid-*β* oligomers (oAβ) and tested the presumptive neuroprotective effect of mechanical force.

## 2 Materials and methods

### 2.1 Ethical statement

Animal procedures were performed in strict compliance with protocols approved by the Italian Ministry of Public Health and the local Ethics Committee of the University of Pisa, in accordance with the European Directive for the Care and Use of Animals 2010/63/EU (project license no. 39E1C.N.5Q7 approved on 30/10/2021). C57BL/6 J mice were used. Animals were maintained in a regulated environment (23 ± 1°C, 50% ± 5% humidity) with a 12-h light–dark cycle and food and water *ad libitum*. The here described hiPSCs are commercially available,^1^ in several modifications, and are listed in several registries (e.g., https://hpscreg.eu/cell-line/UCSFi001-A). It is thus an established and commercially available cell line.

### 2.2 Cell culture

For primary mouse HNs culture, P0/P1 (postnatal day 0/1) stage mice were sacrificed and both hippocampi were explanted and dissected in a solution of ice-cold 6.5 mg/mL D-glucose in Dulbecco’s Phosphate-Buffered Saline (DPBS, Gibco, #14190-144). Tissue was digested for 10 min in 0.25% Trypsin (Gibco, #15050-065) at 37°C and for 5 min in 0.25% Trypsin added with 1% DNase 10 mg/mL (Sigma-Aldrich, #DN25) at 37°C. After centrifugation at 1,200 rpm for 1 min, mechanical dissociation was carried out by passing the cells through a Pasteur glass pipette (VWR, #612-1702) 30 to 40 times. Cells were seeded at cell density of 50,000 cells/cm^2^ in high glucose Dulbecco’s Modified Eagle Medium (DMEM, Gibco, #21063-029) modified with 10% fetal bovine serum (FBS, Gibco, #10270-106), 100 IU/mL penicillin, 100 μg/mL streptomycin (Gibco, #15140-122), and GlutaMAX supplement (Gibco, #35050-038). Cells were seeded on 13 mm circular plastic coverslips (Sarstedt, #83.1840.002) pre-coated with 100 μg/mL Poly-L-lysine (PLL, Sigma-Aldrich, #P4707) and incubated at 37°C in a saturated humidity atmosphere containing 95% air and 5% CO_2_. The medium was replaced at day *in vitro* 0 (*DIV0*) 4 h after seeding with Neurobasal-A medium (Gibco, #12349-015) modified with B27 supplement (Gibco, #17504-044), GlutaMAX, 50 IU/mL penicillin, 50 μg/mL streptomycin, and 5 μg/mL MNPs.

Human cortical neurons were derived from hiPSC line Ngn2-WTC11 as generated in (38). hiPSCs were routinely cultured in StemMACS iPS-Brew-XF medium (Miltenyi Biotec, #130-104-368) supplemented with Antibiotic-Antimycotic Solution (Merck, #A5955) on plates pre-coated with 200 μg/mL Geltrex (Thermo Fisher Scientific, #A1413302) in KnockOut DMEM (KO-DMEM, Thermo Fisher Scientific, #10829018). Cells were incubated at 37°C in a saturated humidity atmosphere containing 95% air and 5% CO_2_ and regularly passaged when 80% confluent in a 1:10 ratio, using Versene (Thermo Fisher Scientific, #15040066) and StemMACS iPS-Brew-XF supplemented with 2 μM thiazovivin (Axon Medchem, #Axon1535) for the first 24 h. For pre-differentiation, on the first day (*DIV0*), cells were incubated in Accutase (Sigma-Aldrich, #A6964) for 5 min at 37°C; then, DPBS was added, and cells were centrifuged at 400 g for 5 min. Supernatant was discarded, and the pellet was re-suspended in StemMACS iPS-Brew-XF. Cells were seeded onto Geltrex-coated plates in pre-differentiation medium consisting of KO-DMEM/F12 (Thermo Fisher Scientific, #12660012) containing N2 supplement (Pan-Biotech, #P07-11010), non-essential amino acids (NEAA, Thermo Fisher Scientific, #11140035), 1.5 μg/mL mouse laminin (Sigma, #L2020), 10 ng/mL brain-derived neurotrophic factor (BDNF, Peprotech, #450-02), 10 ng/mL neurotrophin-3 (NT3, Peprotech, #450-03), 2 μM thiazovivin, 2 μg/mL doxycycline (Merck, #D9891), and Antibiotic-Antimycotic solution. The medium was changed daily for 2 days to fresh pre-differentiation medium without thiazovivin. The duration of the pre-differentiation protocol is 3 days (from *DIV0* to *DIV2*). Hereafter, we refer to these cells as induced neurons (iN) when the precursors have completed the pre-differentiation protocol and are at any stage of terminal differentiation (24 ≥ *DIV*≥3). For terminal differentiation (*DIV3*), cells were dissociated as described for pre-differentiation and seeded onto 13 mm circular plastic coverslips pre-coated with 20 μg/mL Poly-D-Lysine (Sigma-Aldrich, #P7886) and 20 μg/mL Cultrex 3-D Laminin I (Biotechne, #3446-005-01) in maturation medium consisting of 50% DMEM/F12 (Thermo Fisher, #11320033), 50% Neurobasal-A, B27 supplement, N2 supplement, GlutaMax, NEAA, 1.5 μL/mL mouse laminin, 10 ng/mL BDNF, 10 ng/mL NT3, 2 μg/mL doxycycline, and Antibiotic-Antimycotic solution at a seeding density of 50,000 cells/cm^2^. The medium was replaced 4 h after seeding by maturation medium supplemented with 5 μg/mL MNPs.

For both cell cultures, the coverslips were placed inside 35 mm Petri dishes (VWR, #734-2317) that are compatible with the insertion inside the magnetic applicator.

### 2.3 Magnetic nanoparticles

The MNPs used in this study are the FluidMAG-ARA (Chemicell, #4115). They are characterized by an organic iron oxide core with a diameter of 75 ± 10 nm and a magnetic saturation of 59 Am^2^/Kg, as stated from the supplier. They are coated with a polysaccharide shell composed of glucuronic acid, making the hydrodynamic diameter approximately 100 nm. MNPs were added to the cell growth medium at a concentration of 5 μg/mL.

### 2.4 Magnetic field and application of mechanical force

Twenty-four hours after seeding, an external static magnetic field was applied to the cell cultures by placing the 35 mm Petri dishes within a toroidal magnetic applicator Halbach-type that provides a constant magnetic field gradient (46.5 T/m) in the radial centrifugal direction (27, 39). Cells were maintained inside the magnet for 48 h of stimulation.

### 2.5 Neurotoxic oligomer preparation and treatment

For oTau preparation, 1 μM recombinant human hT40 protein (TAU441, Abcam, #ab191460) was incubated with 18.75 μM arachidonic acid (Cayman Chemicals, #90010.1) for 15 min at room temperature (RT) in a polymerization buffer consisting of 10 mM HEPES pH 7.6, 100 mM NaCl, 5 mM DTT, and 0.1 mM EGTA. The solution was stored at −80° *C.* prior to treatment, the solution was centrifuged at 1,400*g* for 5 min; then, oTau were administered to primary mouse HNs in conditioned growth medium at a final concentration of 50 nM, 24 h after seeding (*DIV1*).

For oAβ preparation, Aβ40 and Aβ42 powder (21st Century Biochemicals, #AB40-0010 and #AB42-0010) were dissolved in Hexafluoro-2-propanol (HFIP, 100%) to 1 mM concentration. Then, the HFIP was completely evaporated overnight (ON) in a Speedvac centrifugational evaporator, and the lyophilized powder aliquots were stored at −80°C. For reconstitution, directly before use, dried aliquots were dissolved in 50 mM NaOH; then, Aβ40 and Aβ42 were mixed in a 7:3 ratio and added with DPBS and 50 mM HCl obtaining a final Aβ concentration of 100 μM. To induce oAβ formation, the Aβ mixture was incubated at 37°C for 1 h; then, oAβ were administered to hiPSC-derived cortical iNs in conditioned maturation medium at a final concentration of 1 μM, 24 h and 48 h after seeding (Day 1 and Day 2 of terminal differentiation).

### 2.6 Immunostaining

At *DIV3* for HNs and at *DIV6* for iNs, cells were fixed, immunostained, and imaged.

For HNs, after two washes with DPBS, cells were fixed in 2% w/v paraformaldehyde (PFA) and 7.5% w/v sucrose (Sigma-Aldrich, #S0389) in DPBS at RT for 20 min. Samples were washed three times with DPBS and permeabilized in 0.5% v/v Triton X-100 (Sigma-Aldrich, #X100) in DPBS at RT for 10 min. After three 3-min washes in 0.1% v/v Triton X-100 in DPBS, cells were blocked in 5% v/v goat serum (Gibco, #16210-064) and 0.3% v/v Triton X-100 in DPBS for 1 h at RT and incubated with the primary antibodies ON at 4°C in 3% v/v goat serum and 0.2% v/v Triton X-100 in DPBS. After ON incubation, samples were washed and incubated with secondary antibodies and Hoechst 33342 (Invitrogen, #H3570, dilution 1:1,000) in 3% v/v goat serum and 0.2% v/v Triton X-100 in DPBS and then washed three times in DPBS.

For iNs, after one wash in DPBS, cells were fixed in 3.7% w/v PFA and 4% sucrose in DPBS at RT for 1 h. After three washes in PBS, cells were permeabilized and blocked in 0.5% v/v Triton X-100 and 5% w/v BSA (Carl Roth, #8076.4) in DPBS at RT for 5 min. Cells were washed once in DPBS and incubated with the primary antibodies ON at 4°C in DPBS. After ON incubation, samples were washed three times in DPBS and incubated with secondary antibodies in DPBS for 2 h at RT. After two washes in DPBS, samples were incubated with NucBlue (Thermo Fisher Scientific, #R37605, dilution 1:1,000) in DPBS for 10 min at RT and washed once in deionized water.

The primary antibody used for axon length analysis is Anti-*β*-Tubulin III (TUBBIII, Sigma-Aldrich, #T8578, dilution 1:500). The primary antibodies used for MT stability analysis are Anti-acetylated *α*-tubulin (Sigma-Aldrich, #T7451, dilution 1:400) and Anti-tyrosinated α-tubulin [YL1/2] (Abcam, #ab6160, dilution 1:400). The secondary antibodies used are Thermo Fisher Scientific #A11029, #R6393, and #A11077 (dilution 1:1000).

Coverslips were mounted onto glass microscope slides (Epredia, #AA00000102E01MNZ10) using Aqua-Poly/Mount (Polysciences, #18606-20) and left to dry ON at RT protected from light.

### 2.7 Imaging

In HNs, cell imaging for analysis of axon and dendrite length and pyknotic nuclei was performed at 10X magnification using an inverted fluorescence microscope (Nikon Eclipse Ti) equipped with Nikon DS-Ri2 camera and the help of NIS-Elements AR software version 5.11; cell imaging for analysis of MT stability was performed at 60X magnification with a laser scanning confocal microscope (Nikon AX) and the help of NIS-Elements software version 5.42.

In iNs, cell imaging for analysis of neural processes length and pyknotic nuclei was performed at 20X magnification using a fluorescence microscope (Zeiss Axioscope 5) and the ZenBlue Pro imaging software version 2.5; cell imaging for analysis of MT stability was performed using the same microscope and software, at 40× magnification.

### 2.8 Images analysis

Images were analyzed with Fiji software version 1.54i.

Length of TUBBIII-positive axons and dendrites was measured, and the number of dendrites per cell was counted manually using the NeuronJ plugin. A total of 30 non-interconnected axons per replicate were traced using the tracing tool, and their length data were collected. A total of 30 cells per replicate were used for dendrites analysis, and the average length and number of dendrites per cell data were collected.

For oligomer neurotoxicity testing, cell death was calculated as the percentage of pyknotic nuclei per replicate, analyzing at least 2,000 total nuclei or six pictures per replicate. The status (pyknotic/non-pyknotic) of nuclei was evaluated by the operator considering the level of brightness and compactness. Pyknotic and total nuclei in a picture were counted with Fiji’s CellCounter tool.

For MT stability analysis, the intensities of fluorescent signals of acetylated and tyrosinated *α*-tubulin were quantified as mean fluorescence ( $\bar{f}$ ). Mean fluorescence ( $\bar{f}$ ) of a specific channel in a specific region of interest (ROI) was calculated as follows:

$$\bar{f}=\frac{\text{IntDen}-({\bar{f}}_{\text{back}}\cdot A)}{A}$$

where IntDen is the integrated density (the sum of the intensities of all the pixels in the ROI), ${\bar{f}}_{\text{back}}$ is the mean fluorescence of background readings, and A is the area of the ROI. All these parameters are measured by Fiji software. As a ROI, non-interconnected axons, dendrites, or somas were selected. For background readings, three circular ROIs were selected in close proximity to the analyzed axons/dendrites/somas and their fluorescence values were averaged. At least 12 cells per replicate were analyzed. Stability was calculated as the ratio between acetylated versus tyrosinated *α*-tubulin mean fluorescence.

### 2.9 Statistical analysis

For elongation and MT stability, *a priori* power analysis for required sample size computation was performed with GPower 3.1 software (input parameters: α = 0.05, *β* = 0.20, two-tailed hypothesis, effect size calculated from pilot study data). Data were plotted, and statistical analysis was performed using GraphPad Prism software, version 8.0.2. Values are reported as mean ± standard error of the mean (SEM) from four replicates. Data distribution was checked through different normality tests: Anderson-Darling, D’Agostino & Pearson, Shapiro–Wilk, and Kolmogorov–Smirnov. Specifically, when data did not show a Gaussian distribution, Mann–Whitney test was used for comparing two groups and Kruskal–Wallis test followed by Dunn’s multiple comparisons test was used for comparing three groups. When data showed a Gaussian distribution, *t*-test was used for comparing two groups and one-way analysis of variance (ANOVA) followed by Holm-Sidak’s multiple comparisons test was used when comparing three groups. For the comparison of the frequency distribution of dendrites number per cell, chi-squared test was applied. *p*-values of ≤ 0.05 were considered statistically significant.

The positive control (nano-pulling) is not plotted, but it is reported for the dataset related to iNs in Supplementary Figure S2.

## 3 Results

### 3.1 AD-related Tau pathology stressors cause axonal shortening in *in vitro* models

Although various forms of Aβ and Tau aggregates have been shown to exhibit some level of toxicity, oligomers are currently considered to be the most harmful (40, 41). For this reason, we used oligomeric species of Tau and Aβ as stressors in our models. For Tau oligomerization, we modified the protocol from Caneus et al. (41), optimizing the concentration, 50 nM, that is able to induce a 30% shortening of axon length in mouse HNs (Supplementary Figure S1). For Aβ oligomerization, we used an oligomeric preparation of a 7:3 ratio of Aβ40/Aβ42 at 1 μM concentration since we had previously reported that it vigorously induces hallmarks of AD (17). As this oligomers mix shows reversion of toxicity after 24 h (17), treatment was repeated for 2 consecutive days.

**Figure 1:**
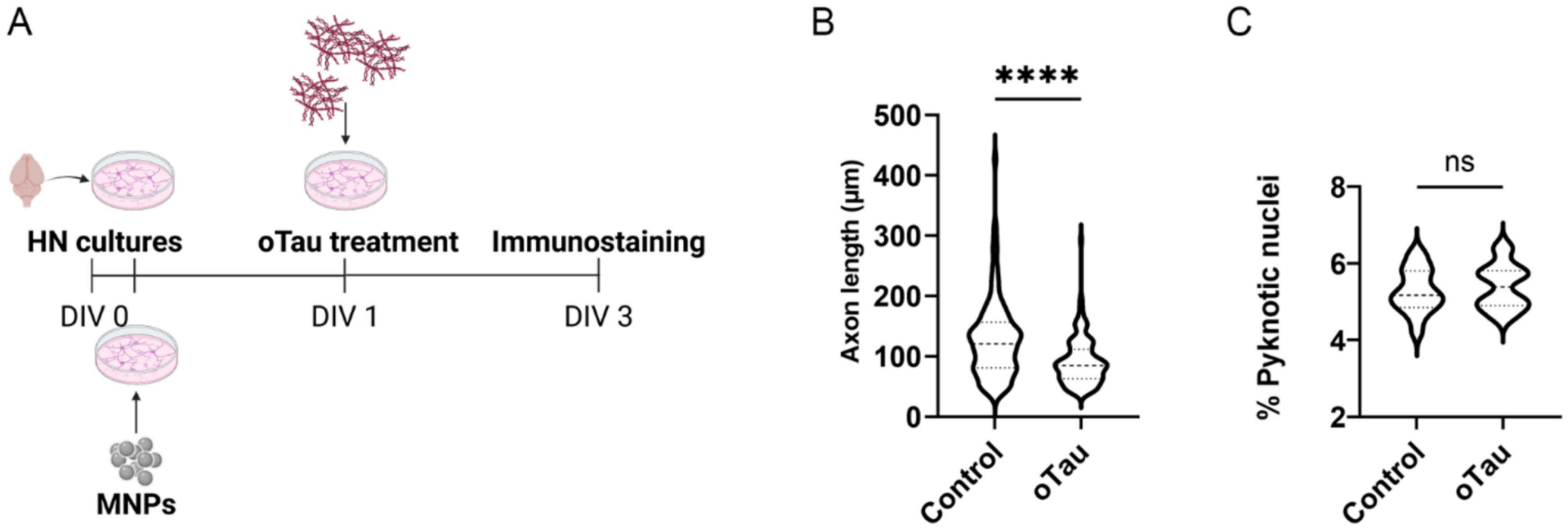
Cell model of AD-related Tau pathology: effects of oTau on primary mouse HNs. **(A)** Schematic representation of the protocol. **(B)** Elongation analysis of primary mouse HNs treated with 50 nM oTau versus control condition. Violin plot, data are expressed as median (dashed line) and 25–75 percentiles (dotted lines). *n* = 120, from four replicates. Mann–Whitney test for unpaired data, two-tailed. **** = *p* < 0.0001. **(C)** Analysis of the percentage of pyknotic nuclei of primary mouse HNs treated with 50 nM oTau versus control condition. Violin plot, data are expressed as median (dashed line) and 25–75 percentiles (dotted lines). *n* = 16, from four replicates. *N* > 2000 of total nuclei for each replicate. Unpaired *t*-test, two-tailed. ns, not significant.

HNs were treated with 50 nM oTau at *DIV1,* and analysis was performed at *DIV3* (Figure 1A). To evaluate the detrimental effect of the neurotoxic oligomers, we measured the axonal length, already used in literature as an indicator of neuron functionality (42). A statistically significant delay of outgrowth of axons in the treated samples compared to control samples was observed. The axon length reduction obtained by administering 50 nM oTau to mouse HNs is 28.6% [average axon length was 127.8 ± 5.8 μm in controls and 91.3 ± 3.6 μm in oTau-treated samples, *p* < 0.0001 (Figure 1B)]. The average percentage of cell death was 5.3 ± 0.14% in controls and 5.4 ± 0.14% in treated samples (*p* = 0.32, Figure 1C).

**Figure 2:**
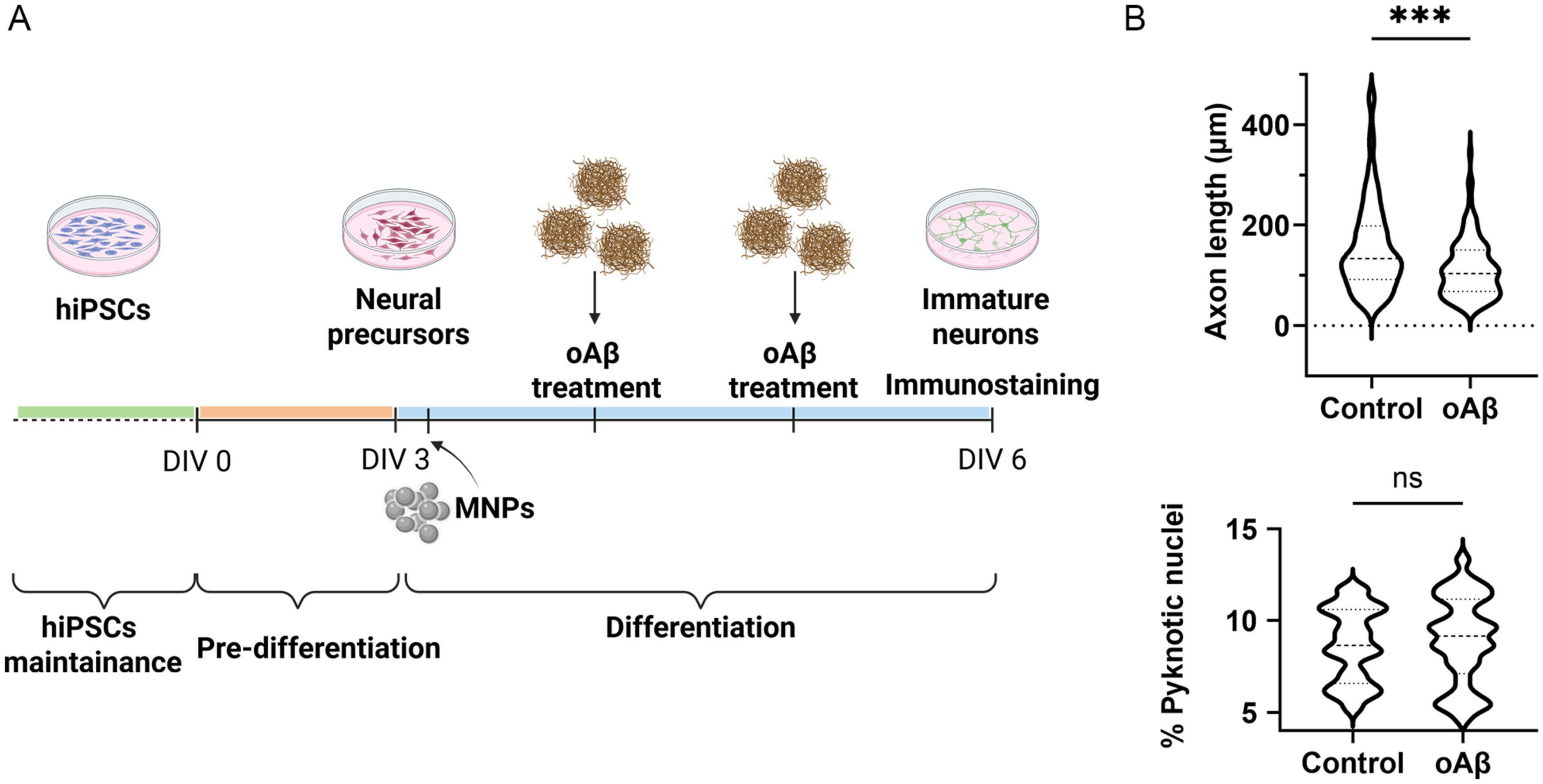
Cell model of AD: effects of oAβ on hiPSC-derived cortical iNs. **(A)** Schematic representation of the protocol. **(B)** Elongation analysis of hiPSC-derived cortical iNs treated with 1 μM oAβ versus control condition. Violin plot, data are expressed as median (dashed line) and 25–75 percentiles (dotted lines). *n* = 120, from four replicates. Mann–Whitney test for unpaired data, two-tailed. *** = *p* < 0.001. **(C)** Analysis of the percentage of pyknotic nuclei of hiPSC-derived cortical iNs treated with 1 μM oAβ versus control condition. Violin plot, data are expressed as median (dashed line) and 25–75 percentiles (dotted lines). *n* = 24, from four replicates. *N* > 1,000 of total nuclei or at least six pictures analyzed for each replicate. Unpaired *t*-test, two-tailed. ns, not significant.

iNs were treated with 1 μM oAβ at *DIV4,* and analysis was performed at *DIV6* (Figure 2A). Similarly to the previous model, a reduction in the length of the neural processes was obtained. The reduction obtained was 24.6% [the average length of neural processes was 155.3 ± 8.2 μm in controls and 113.4 ± 5.3 μm in oAβ-treated samples, *p* = 0.0002 (Figure 2B)], and the average percentage of cell death was 8.7 ± 0.4% in controls and 8.9 ± 0.4% in treated samples (*p* = 0.65, Figure 2C).

These results show that our treatments impact process elongation but not cell viability, mimicking the first stage of pathology.

### 3.2 Nano-pulling compensates for axonal shortening in cell models subjected to AD-related Tau pathology stressors

To assess the neuroprotective effect of mechanical stimulation, three experimental groups were established: a control group that provides the baseline and reference values of the analyzed parameters, a group treated with oligomers from which we expect a decline in these values, and a group treated with oligomers and subjected to mechanical stimulation, from which we expect a recovery of the control values if the nano-pulling exerts a neuroprotective effect.

**Figure 3:**
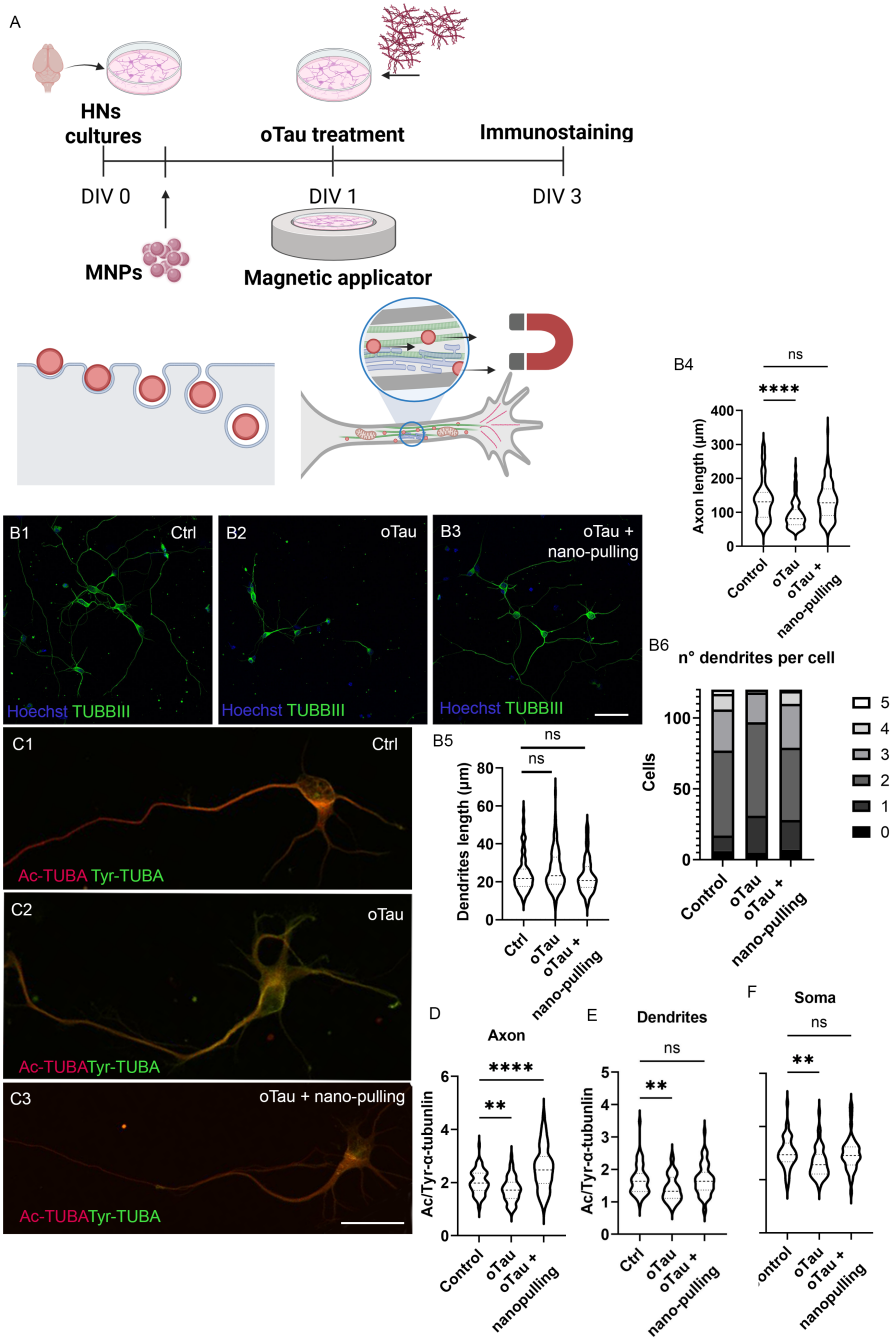
Effect of nano-pulling on primary mouse HNs treated with oTau. **(A)** Schematic representation of the protocol. Nano-pulling is a technique that consists in adding MNPs to neurons (DIV0). The particles are internalized and accumulate in cell cytoplasm, including the axoplasm, where they interact with organelles and cytoskeleton. When external magnetic applicator is applied (DIV1), a stretching force inside the axon is generated. **(B1–B3)** Representative pictures of mouse HNs subjected to the different experimental conditions and stained for TUBBIII (green) and Hoechst (blue). Ctrl = control group, oTau = pathological group, oTau + nano-pulling = pathological group simultaneously subjected to nano-pulling. Scale bar corresponds to 50 μm. **(B4)** Axon elongation analysis of primary mouse HNs treated with 50 nM oTau and simultaneously “stretched” with mechanical stimulation. Violin plot, data are expressed as median (dashed line) and 25–75 percentiles (dotted lines). *n* = 120, from four replicates. Kruskal–Wallis test for unpaired data followed by Dunn’s multiple comparisons test, two-tailed. Mean rank of each group is compared with mean rank of control group. **** *p* < 0.0001, ns, not significant. **(B5)** Dendrite elongation analysis of primary mouse HNs treated with 50 nM oTau and simultaneously “stretched” with mechanical stimulation. Violin plot, data are expressed as median (dashed line) and 25–75 percentiles (dotted lines). *n* = 120, from four replicates. Kruskal–Wallis test for unpaired data followed by Dunn’s multiple comparisons test, two-tailed. Mean rank of each group is compared with mean rank of control group. ns, not significant. **(B6)** Dendrite number per cell in primary mouse HNs treated with 50 nM oTau and simultaneously “stretched” with mechanical stimulation. Contingency plot. *n* = 120 cells, from four replicates. Chi-squared test. Control vs. oTau: Chi-square = 16.97, df = 5, *p* = 0.005. Control vs. oTau+nano-pulling: Chi-square = 5.198, df = 5, *p* = 0.39. **(C1–C3)** Representative pictures of mouse HNs subjected to the different experimental conditions and stained for Ac-α-tubb (red) and Tyr-α-tubb (green). Ctrl = control group, oTau = pathological group, oTau + nano-pulling = pathological group simultaneously subjected to nano-pulling. Scale bar corresponds to 25 μm. **(D)** Axonal MT stability analysis of HNs treated with 50 nM oTau and simultaneously “stretched” with mechanical stimulation. Violin plot, data are expressed as median (dashed line) and 25–75 percentiles (dotted lines). *n* = 70 from four replicates. One-way ANOVA test for unpaired data followed by Holm-Sidak’s multiple comparisons test, two-tailed. Mean rank of each group is compared with mean rank of control group. **** *p* < 0.0001 ** *p* < 0.01. **(E)** MT stability analysis in dendrites of HNs treated with 50 nM oTau and simultaneously “stretched” with mechanical stimulation. Violin plot, data are expressed as median (dashed line) and 25–75 percentiles (dotted lines). *n* = 60 from four replicates. Kruskal–Wallis test for unpaired data followed by Dunn’s multiple comparisons test, two-tailed. Mean rank of each group is compared with mean rank of control group. ** *p* < 0.01, ns, not significant. **(F)** Somatic MT stability analysis of HNs treated with 50 nM oTau and simultaneously “stretched” with mechanical stimulation. Violin plot, data are expressed as median (dashed line) and 25–75 percentiles (dotted lines). *n* = 50 from four replicates. Kruskal–Wallis test for unpaired data followed by Dunn’s multiple comparisons test, two-tailed. Mean rank of each group is compared with mean rank of control group. ** *p* < 0.01. ns, not significant.

For HNs, MNPs were applied 4 h after cell isolation and seeding, oligomer treatment was performed at *DIV1,* and the mechanical stimulation was performed from *DIV1* to *DIV3* (Figure 3A). The analysis of axon length at *DIV3* showed that the reduction in the axon length due to oTau treatment was recovered when the cells were subjected to nano-pulling (Figures 3B1, 3B2, 3B3). Specifically, the average axon length in the control group was 134.6 ± 5.4 μm, in the pathological group was 92.3 ± 3.6 μm, and in the pathological group subjected to mechanical stimulation was 133.8 ± 4.9 μm (Figure 3B4) (*p* < 0.0001 between the pathological and control groups and *p* = 0.18 between the pathological group subjected to nano-pulling and the control group).

Given that Tau pathology primarily affects dendrites, we also evaluated the effects of oTau and nano-pulling in this compartment. We found that dendrite length is not influenced by oTau nor oTau in combination with nano-pulling (specifically, the average dendrite length in the control group was 22.93 ± 0.74 μm, in the pathological group was 25.65 ± 0.90 μm, and in the pathological group subjected to mechanical stimulation was 22.91 ± 0.85 μm, *p* = 0.1 between the pathological and control groups, and *p* = 1 between the pathological group subjected to nano-pulling and the control group) (Figure 3B5). However, the study of the frequency distribution of the dendrite number per cell shows a reduction by oTau treatment with respect to the control (*p* = 0.005) that is compensated when cells are simultaneously subjected to nano-pulling (*p* = 0.39 versus control) (Figure 3B6).

**Figure 4:**
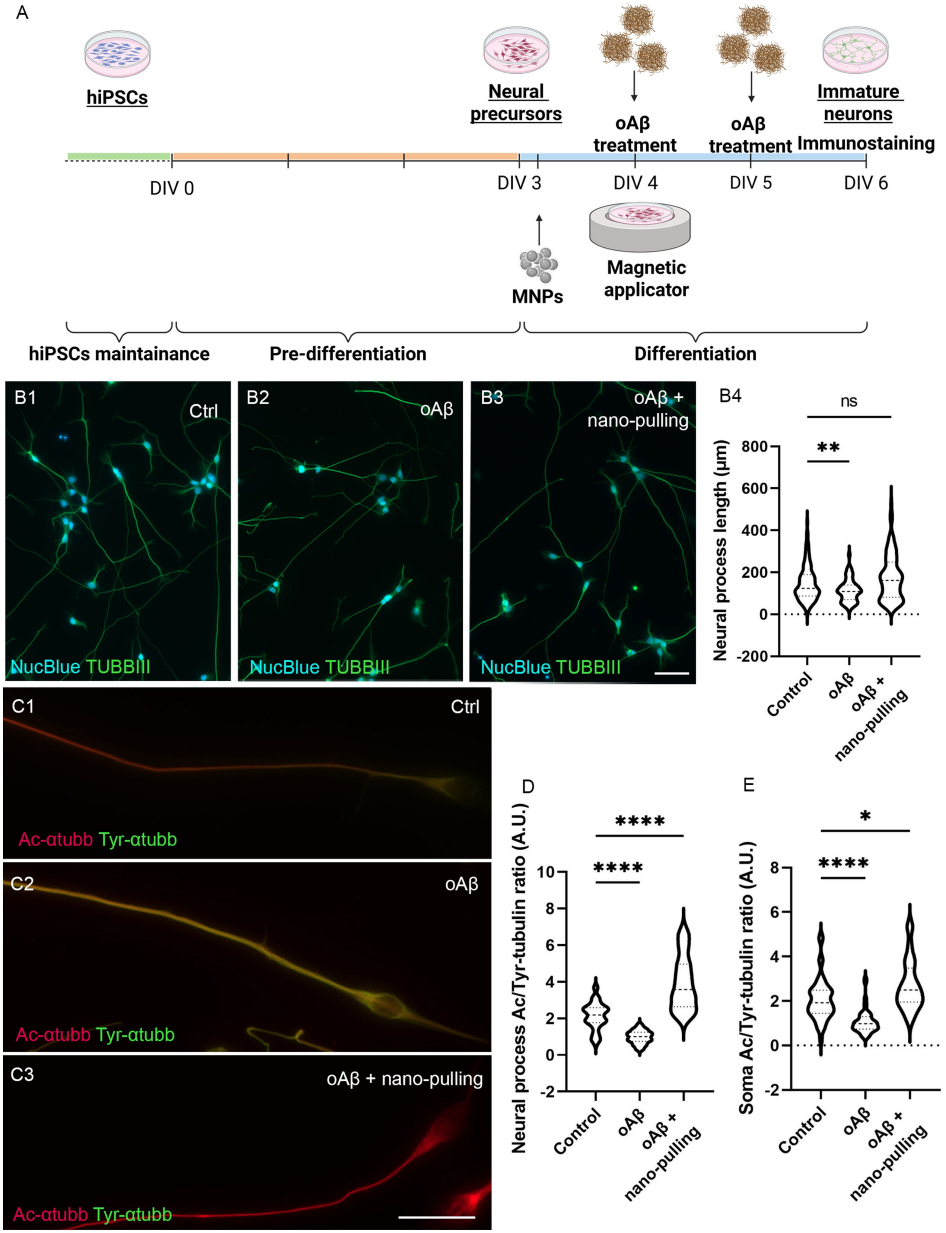
Effect of nano-pulling on hiPSC-derived cortical iNs treated with oAβ**. (A)** Schematic representation of the protocol. **(B1–B3**) Representative pictures of hiPSC-derived cortical iNs subjected to the different experimental conditions and stained for TUBBIII (green) and NucBlue (cyan). Ctrl = control group, oAβ = pathological group, oAβ + nano-pulling = pathological group simultaneously subjected to nano-pulling. Scale bar corresponds to 50 μm. **(B4)** Elongation analysis of hiPSC-derived cortical iNs treated with 1 μM oAβ and simultaneously “stretched” with mechanical stimulation. Violin plot, data are expressed as median (dashed line) and 25–75 percentiles (dotted lines). *n* = 120, from four replicates. Kruskal–Wallis test for unpaired data followed by Dunn’s multiple comparisons test, two-tailed. Mean rank of each group is compared with mean rank of the control group. ** *p* < 0.01, ns, not significant. **(C1–C3)** Representative pictures of hiPSC-derived cortical iNs subjected to the different experimental conditions and stained for Ac-α-tubb (red) and Tyr-α-tubb (green). Ctrl = control group, oAβ = pathological group, oAβ + nano-pulling = pathological group simultaneously subjected to nano-pulling. Scale bar corresponds to 25 μm. **(D)** Analysis of MT stability in neural processes of iNs treated with 1 μM oAβ and simultaneously “stretched” with mechanical stimulation. Violin plot, data are expressed as median (dashed line) and 25–75 percentiles (dotted lines). *n* = 48 from four replicates. Kruskal–Wallis test for unpaired data followed by Dunn’s multiple comparisons test, two-tailed. Mean rank of each group is compared with mean rank of the control group. **** *p* < 0.0001. **(E)** Somatic MT stability analysis of iNs treated with 1 μM oAβ and simultaneously “stretched” with mechanical stimulation. Violin plot, data are expressed as median (dashed line) and 25–75 percentiles (dotted lines). *n* = 50 from four replicates. Kruskal–Wallis test for unpaired data followed by Dunn’s multiple comparisons test, two-tailed. Mean rank of each group is compared with mean rank of the control group. **** *p* < 0.0001 * *p* < 0.05.

For iNs, MNPs were applied 4 h after the beginning of terminal differentiation (*DIV3*), oligomer treatment was performed at *DIV4,* and the mechanical stimulation was performed from *DIV4* to *DIV6* (Figure 4A). A similar effect was observed on the neural processes of iNs: The average length in the control group was 147.4 ± 7.6 μm, in the pathological group was 113.7 ± 4.9 μm, and in the pathological group subjected to mechanical stimulation was 175.9 ± 9.9 μm (Figure 4B) (*p* = 0.009 between the pathological and control groups and *p* = 0.18 between the pathological group subjected to nano-pulling and the control group).

These data show that mechanical stimulation with piconewton forces prevents axonal shortening induced by oTau and oAβ, restoring outgrowth to normal physiological levels.

### 3.3 Nano-pulling overcompensates for MT destabilization in cell models subjected to AD-related Tau pathology stressors

Using the same experimental design, to assess whether the neuroprotective effect of mechanical stimulation is mediated by force-induced MT stabilization, we evaluated the stability of axonal MTs in the two models. Cells were co-immunostained for acetylated and tyrosinated *α*-tubulin, routinely used markers of stable and dynamic MTs, respectively (43–45), and the ratio between the two fluorescent signals was used as a measure of MT stability (Figure 3C). Analysis was performed at *DIV3* for HNs and at *DIV6* for iNs (Figure 4C).

Data analysis showed that in HNs treated with oTau in the absence of mechanical stimulation, there is a statistically significant destabilization of MTs in the axons compared to the control group (2.02 ± 0.05 and 1.74 ± 0.06 for control and oTau, respectively, *p* = 0.0065), while in presence of mechanical stimulation, there is a statistically significant increase of the stability compared to the control group (2.02 ± 0.05 and 2.55 ± 0.1 for control and oTau+nano-pulling, respectively, *p* < 0.0001) (Figure 3D). Similarly, in iNs treated with the oAβ in absence of mechanical stimulation, there is a statistically significant destabilization of MTs in the neural projections compared to the control group (2.13 ± 0.11 and 0.98 ± 0.05 for control and oAβ, respectively), while in presence of mechanical stimulation, there is a statistically significant increase of the stability compared to the control group (2.13 ± 0.11 and 3.93 ± 0.21 for control and oAβ + nano-pulling, respectively, *p* < 0.0001) (Figure 4D). This result suggests that oTau and oAβ are able to induce MT destabilization at the axonal level and nano-pulling not only compensates this loss but also increases MT stability over physiological levels, acting as a neuroprotective factor.

We also evaluated whether the stabilizing effect propagates to MT in the cell soma, using the same experimental design. Data analysis showed that in cells treated with the neurotoxic oligomers in the absence of mechanical stimulation, there is a statistically significant destabilization of MTs in the somas compared to the control group. In particular, in the HNs model, the ratio between the mean fluorescence of acetylated and tyrosinated *α*-tubulin found in somas of controls was 1.50 ± 0.04, while in samples treated with 50 nM oTau it was 1.32 ± 0.04 (*p* = 0.0017) (Figure 3F). In the iNs model, the ratio was 2.05 ± 0.13 in controls and 1.06 ± 0.07 in samples treated with 1 μm oAβ (*p* < 0.0001). Mechanical stimulation was able to restore the soma MT stability levels of control condition in the HNs model (ratio 1.49 ± 0.04, *p* = 1 compared to the control group) (Figure 3F). In the soma of oAβ-treated iNs, nano-pulling was able to increase the level of MT stability beyond that of the control group (ratio 2.71 ± 0.17, *p* = 0.02 compared to the control group) (Figure 4E). This result suggests that oTau and oAβ also induce MT destabilization at the soma level and nano-pulling is able to compensate for induced destabilization, restoring or overcompensating MT stability levels in soma, depending on the cell model.

Similarly in dendrites of HNs, MT stability is reduced by oTau treatment and compensated in the group simultaneously subjected to nano-pulling: the ratio between the mean fluorescence of acetylated and tyrosinated *α*-tubulin found in dendrites of controls was 1.68 ± 0.06, in samples treated with 50 nM oTau was 1.42 ± 0.05 (*p* = 0.0029 versus control), and in treated samples simultaneously subjected to nano-pulling was 1.68 ± 0.48 (*p* = 1 versus control) (Figure 3E). These results suggest that oTau also induce damages at the dendrites level (consisting in reduction in number and MT destabilization) and nano-pulling is able to compensate, restoring dendrites number and MT stability to a physiological level.

## 4 Discussion

In tauopathies, Tau sequestration into neurofibrillary tangles induces MT destabilization, causing alterations in axonal transport and eventually leads to neurite collapse (46). In light of this, researchers have proposed MT stabilization in neurons through exogenous factors as a potential therapeutic strategy for these pathologies, based on a functional substitution of Tau (47). Pharmacological approaches have already been tested, both *in vivo* and *in vitro*, using MT-stabilizing compounds that in most cases are already used as anti-cancer drugs (48). In *in vitro* models, these drugs have been found to rescue degenerating primary neurons in the presence of neurotoxicity induced by amyloid-*β* (49) or Tau (50). In transgenic animal models of tauopathy, they were able to reverse axonal transport deficits (51), improve cognitive deficits (52, 53), reduce neurons loss (54), and reverse spine changes (55–57). More recently, these drugs showed reduction in molecular markers of pathology, cognitive improvement, and mitigation of synaptic pathology also in transgenic animal models of AD (58–60). These findings prove that MT stabilization can compensate for the loss of Tau function and its detrimental effects in AD-related Tau pathology, demonstrating the therapeutic potential of this approach. However, MT-stabilizing agents are widely used in clinics as chemotherapeutic drugs, and there are many side effects associated with their use (61), limiting their translational application to neurodegenerative disorders. In this study, we explored the use of mechanical force as an alternative extrinsic factor to stabilize MTs (11). Specifically, the nano-pulling technique developed by our group for chronical mechanical stimulation of neurons *in vitro* (39) has been shown to stimulate MT stabilization (27, 29, 32, 34) and induce differential expression of genes associated with MT cytoskeleton organization, MT-binding proteins, and MT motor protein. More importantly, we extensively tested nano-pulling in primary neurons (HNs, dorsal root ganglion neurons) (29, 32, 33), NPCs (34), and spinal cord tissue (34), and no signs of toxicity have ever been reported.

In this context, we hypothesized that nano-pulling could be exploited as a novel neuroprotective strategy for the prevention or reduction in MT instability and its neurotoxic consequences in paradigms of tauopathy. We here aimed to test its effects in *in vitro* models of amyloid-*β* or Tau-oligomers induced neurotoxicity. Several studies have shown that oligomers can be internalized and that this is likely an important contributor to the neurotoxic effects. The process typically happens through receptor-mediated endocytosis or clathrin-mediated pathways (62, 63). Despite this, not all oligomers are immediately internalized: Some interact with membrane receptors, such as metabotropic glutamate (mGluR) receptors or N-methyl-D-aspartate receptor (NMDAR), altering signaling at the cell surface before endocytosis (64, 65). Specifically, we decided to treat iNs with oAβ because, in our previous studies, we found that iPSC-derived human neurons exhibited profound changes when exposed to oligomeric Aβ (e.g., Tau missorting, loss of microtubule stability, decreased neurotransmission as assessed by calcium imaging, and reduced synaptic density) but not in response to the aggregation/oligomerization-prone P301L-TAU (66, 67). On the other hand, considering that the splice-isoform conversion of murine Tau toward more aggregation-prone 4R isoforms occurs at a later developmental stage and earlier in rodents than in humans (68), we opted to test oTau on postnatal murine neurons. Our data support the hypothesis that nano-pulling prevents axonal shortening by acting on MT stability (Figures 3D, 4D). These results are consistent with those coming from pharmacological approaches using MT-stabilizing drugs (48). More interestingly, MT stabilization is an ubiquitous effect that was observed not only in the axons but also in the soma (Figures 3F, 4E) and dendrites (Figure 3E). More specifically, we found that oTau treatment induces a decrease in dendrite sprouting, measured as the number of dendrites per cell, which is prevented when HNs undergo nano-pulling (Figure 3B5). However, neither oTau nor nano-pulling affects dendrite length. This observation is consistent with our proposed model, in which nano-pulling-induced elongation depends on MT stabilization (69). This process occurs in axons—where MTs are preferentially oriented with the + end directed toward the growth cone—when the force is applied from the soma to the tip, but not when it is applied from the tip to the soma (27). Conversely, dendrites—which lack a preferential MT orientation—do not undergo nano-pulling-mediated elongation.

To the best of our knowledge, it is the first time that nano-pulling technique has been applied as a neuroprotective strategy to neurodegeneration models. A limitation of this study is certainly related to the models. In fact oligomers are able to induce detrimental effects such as MT destabilization and processes shortening, being thus useful for modeling certain aspects of the pathology, but are obviously not able to fully mimic all the aspects of the disease and the complexity of the pathological environment characterized by oxidative stress and neuroinflammation. Speculating on a future pre-clinical testing of the methodology in a neurodegeneration setting, nano-pulling could be applied as a minimally invasive strategy consisting in the administration of MNPs to the animal model and the application of a wearable magnetic device to activate the strategy in the area of interest (70). In addition, several approaches involving MNPs have recently been described for crossing the blood–brain barrier and even targeting specific neuronal populations (71–73), making this approach potentially translatable to the treatment of brain neurodegenerative disorders.

## Data Availability

The datasets presented in this study can be found in online repositories. The names of the repository/repositories and accession number(s) can be found at: DOI: 10.5281/zenodo.13987314.

## Footnotes

^1^ https://www.coriell.org/0/Sections/Search/Sample_Detail.aspx?Ref=GM25256

## Supplementary material

SUPPLEMENTARY FIGURE S1 Effects of different concentrations of oTau on primary mouse HNs. Elongation analysis of primary mouse HNs treated with [Tau] = 25 nM, [Tau] = 50 nM, and [Tau]=75 nM versus control condition. Violin plot, data are expressed as median (dashed line) and 25–75 percentiles (dotted lines). *n* = 60, from two replicates. Kruskal-Wallis test for unpaired data followed by Dunn’s multiple comparisons test, two-tailed. Mean rank of each group is compared with mean rank of control group. **** = *p* < 0.0001. A shortening of axons in the treated samples compared to control samples was observed. oTau concentration of 50 nM was chosen, as the lower concentration (25 nM) had no statistically significant effect compared to control neurons, and the higher concentration (75 nM) showed no dose-dependent effect compared to 50 nM.

SUPPLEMENTARY FIGURE S2 Effects of oTau and nano-pulling on iNs—all controls. **(A)** Elongation analysis of hiPSC-derived cortical iNs treated with 1 μM oAβ and subjected to nano-pulling. Violin plot, data are expressed as median (dashed line) and 25–75 percentiles (dotted lines). *n* = 120, from four replicates. Kruskal-Wallis test for unpaired data followed by Dunn’s multiple comparisons test, two-tailed. Interaction: F (1, 476) = 0.3023, *p* = 0.58. Row Factor (oligomer treatment): F (1, 476) = 16.98, *p* < 0.0001. Column Factor (nano-pulling): F (1, 476) = 47.44, *p* < 0.0001. **(B)** Analysis of MT stability in neural processes of iNs treated with 1 μM oAβ and “stretched” with mechanical stimulation. Violin plot, data are expressed as median (dashed line) and 25–75 percentiles (dotted lines). *n* = 48 from four replicates. Kruskal-Wallis test for unpaired data followed by Dunn’s multiple comparisons test, two-tailed. Interaction: F (1, 188) = 5.381, *p* = 0.02. Row Factor (oligomer treatment): F (1, 188) = 25.42, *p* < 0.0001. Column Factor (nano-pulling): F (1, 188) = 275, *p* < 0.0001. **(C)** Somatic MT stability analysis of iNs treated with 1 μM oAβ and “stretched” with mechanical stimulation. Violin plot, data are expressed as median (dashed line) and 25–75 percentiles (dotted lines). *n* = 50 from four replicates. Kruskal-Wallis test for unpaired data followed by Dunn’s multiple comparisons test, two-tailed. Interaction: F (1, 202) = 8.963, *p* = 0.031. Row Factor (oligomer treatment): F (1, 202) = 19.80, *p* < 0.0001. Column Factor (nano-pulling): F (1, 202) = 89.08, *p* < 0.0001.

## Glossary

AD: Alzheimer’s disease
DIV: day *in vitro*
DMEM: Dulbecco’s Modified Eagle Medium
DPBS: Dulbecco’s Phosphate-Buffered Saline
FBS: fetal bovine serum
iN: iPSC-derived neural progenitor cells undergoing neuronal differentiation in cortical neurons
h: human
HFIP: hexafluoro-2-propanol
HN: hippocampal neuron
iPSC: induced pluripotent stem cell
MAP: microtubule-associated protein
MNP: magnetic nanoparticle
MT: microtubule
NFT: neurofibrillary tangles
ns: non-significant
oA*β*: Aβ oligomers
ON: overnight
oTau: Tau oligomers
P: postnatal
PFA: paraformaldehyde
pN: piconewton
ROI: region of interest
RT: room temperature
PLL: poly-L-lysine
TUBBIII: β-tubulin III

## References

[1] Lane CA Hardy J Schott JM. Alzheimer's disease. Eur J Neurol. (2018) 25:59–70. doi: 10.1111/ene.13439, PMID: 28872215

[2] Karran E Mercken M De Strooper B. The amyloid cascade hypothesis for Alzheimer’s disease: an appraisal for the development of therapeutics. Nat Rev Drug Discov. (2011) 10:698–712. doi: 10.1038/nrd3505, PMID: 21852788

[3] Zempel H Thies E Mandelkow E Mandelkow EM. Aβ oligomers cause localized Ca2+ elevation, missorting of endogenous tau into dendrites, tau phosphorylation, and destruction of microtubules and spines. J Neurosci. (2010) 30:11938–50. doi: 10.1523/JNEUROSCI.2357-10.2010, PMID: 20826658 PMC6633549

[4] Jonsson T Atwal JK Steinberg S Snaedal J Jonsson PV Bjornsson S. A mutation in APP protects against Alzheimer’s disease and age-related cognitive decline. Nature. (2012) 488:96–9. doi: 10.1038/nature11283, PMID: 22801501

[5] Shimohama S Fujioka R Mihira N Sekiguchi M Sartori L Joho D. The Icelandic mutation (APP-A673T) is protective against amyloid pathology in vivo. J Neurosci. (2024) 44:e0223242024. doi: 10.1523/JNEUROSCI.0223-24.2024, PMID: 39496485 PMC11580785

[6] Zhang Y Chen H Li R Sterling K Song W. Amyloid β-based therapy for Alzheimer’s disease: challenges, successes and future. Signal Transduct Target Ther. (2023) 8:248. doi: 10.1038/s41392-023-01484-7, PMID: 37386015 PMC10310781

[7] Van Dyck CH Swanson CJ Aisen P Bateman RJ Chen C Gee M. Lecanemab in early Alzheimer’s disease. N Engl J Med. (2023) 388:9–21. doi: 10.1056/NEJMoa2212948 36449413

[8] Braak H Thal DR Ghebremedhin E Del Tredici K. Stages of the pathologic process in Alzheimer disease: age categories from 1 to 100 years. J Neuropathol Exp Neurol. (2011) 70:960–9. doi: 10.1097/NEN.0b013e318232a379, PMID: 22002422

[9] Braak H Del Tredici K. Alzheimer’s disease: intraneuronal alterations precede insoluble amyloid-β formation. Neurobiol Aging. (2004) 25:713–8. doi: 10.1016/j.neurobiolaging.2003.12.015, PMID: 15165692

[10] Blennow K Brody DL Kochanek PM Levin H McKee A Ribbers GM. Traumatic brain injuries. Nat Rev Dis Primers. (2016) 2:16084. doi: 10.1038/nrdp.2016.84, PMID: 27853132

[11] Langerscheidt F Wied T Al Kabbani MA van Eimeren T Wunderlich G Zempel H. Genetic forms of tauopathies: inherited causes and implications of Alzheimer’s disease-like TAU pathology in primary and secondary tauopathies. J Neurol. (2024) 271:2992–3018. doi: 10.1007/s00415-024-12314-3, PMID: 38554150 PMC11136742

[12] Arendt T Stieler JT Holzer M. Tau and tauopathies. Brain Res Bull. (2016) 126:238–92. doi: 10.1016/j.brainresbull.2016.08.018 27615390

[13] Goedert M Spillantini MG. Propagation of tau aggregates Tim bliss. Mol Brain. (2017) 10. doi: 10.1186/s13041-017-0298-7 PMC5450399 28558799

[14] Hirokawa N Niwa S Tanaka Y. Molecular motors in neurons: transport mechanisms and roles in brain function, development, and disease. Neuron. (2010) 68:610–38. doi: 10.1016/j.neuron.2010.09.039 21092854

[15] Baird FJ. Microtubule defects and neurodegeneration. J Genet Syndr Gene Ther. (2013) 4, 203–210. doi: 10.4172/2157-7412.1000203, PMID: 24563812 PMC3930179

[16] Matamoros AJ Baas PW. Microtubules in health and degenerative disease of the nervous system. Brain Res Bull. (2016) 126:217–25. doi: 10.1016/j.brainresbull.2016.06.016, PMID: 27365230 PMC5079814

[17] Zempel H Luedtke J Kumar Y Biernat J Dawson H Mandelkow E. Amyloid-β oligomers induce synaptic damage via tau-dependent microtubule severing by TTLL6 and spastin. EMBO J. (2013) 32:2920–37. doi: 10.1038/emboj.2013.207, PMID: 24065130 PMC3831312

[18] Congdon EE Ji C Tetlow AM Jiang Y Sigurdsson EM. Tau-targeting therapies for Alzheimer disease: current status and future directions. Nat Rev Neurol [Internet]. (2023) 19:715–36. doi: 10.1038/s41582-023-00883-2, PMID: 37875627 PMC10965012

[19] Falconieri A Coppini A Raffa V. Microtubules as a signal hub for axon growth in response to mechanical force. Biol Chem. (2024) 405:67–77. doi: 10.1515/hsz-2023-0173 37674311

[20] Gudimchuk NB Ulyanov EV O’Toole E Page CL Vinogradov DS Morgan G. Mechanisms of microtubule dynamics and force generation examined with computational modeling and electron cryotomography. Nat Commun. (2020) 11:3765. doi: 10.1038/s41467-020-17553-2, PMID: 32724196 PMC7387542

[21] Hamant O Inoue D Bouchez D Dumais J Mjolsness E. Are microtubules tension sensors? Nat Commun. (2019) 10:2360. doi: 10.1038/s41467-019-10207-y, PMID: 31142740 PMC6541610

[22] Franck AD Powers AF Gestaut DR Gonen T Davis TN Asbury CL. Tension applied through the Dam1 complex promotes microtubule elongation providing a direct mechanism for length control in mitosis. Nat Cell Biol. (2007) 9:832–7. doi: 10.1038/ncb1609, PMID: 17572669 PMC2680956

[23] Ciudad A Sancho JM. External mechanical force as an inhibition process in kinesin’s motion. Biochem J. (2005) 390:345–9. doi: 10.1042/BJ20042092, PMID: 15896195 PMC1184588

[24] Molodtsov MI Mieck C Dobbelaere J Dammermann A Westermann S Vaziri A. A force-induced directional switch of a molecular motor enables parallel microtubule bundle formation. Cell. (2016) 167:539–552.e14. doi: 10.1016/j.cell.2016.09.029, PMID: 27716509

[25] Trushko A Schäffer E Howard J. The growth speed of microtubules with XMAP215-coated beads coupled to their ends is increased by tensile force. Proc Natl Acad Sci USA. (2013) 110:14670–5. doi: 10.1073/pnas.1218053110, PMID: 23964126 PMC3767536

[26] Li Y Kučera O Cuvelier D Rutkowski DM Deygas M Rai D. Compressive forces stabilize microtubules in living cells. Nat Mater. (2023) 22:913–24. doi: 10.1038/s41563-023-01578-1, PMID: 37386067 PMC10569437

[27] Raffa V Falcone F De Vincentiis S Falconieri A Calatayud MP Goya GF. Piconewton mechanical forces promote neurite growth. Biophys J. (2018) 115:2026–33. doi: 10.1016/j.bpj.2018.10.009, PMID: 30473016 PMC6303536

[28] Falconieri A De Vincentiis S Radoiffa V. Recent advances in the use of magnetic nanoparticles to promote neuroregeneration. Nanomedicine. (2019) 14:1073–6. doi: 10.2217/nnm-2019-0103 31050591

[29] de Vincentiis S Falconieri A Mainardi M Cappello V Scribano V Bizzarri R. Extremely low forces induce extreme axon growth. J Neurosci. (2020) 40:4997–5007. doi: 10.1523/JNEUROSCI.3075-19.2020, PMID: 32444384 PMC7314409

[30] De Vincentiis S Falconieri A Scribano V Ghignoli S Raffa V. Manipulation of axonal outgrowth via exogenous low 2 forces 3 [internet]. Int J Mol Sci. (2020) 21, 8009–8036. doi: 10.3390/ijms21218009 33126477 PMC7663625

[31] De Vincentiis S Falconieri A Mickoleit F Cappello V Schüler D Raffa V. Induction of axonal outgrowth in mouse hippocampal neurons via bacterial magnetosomes. Int J Mol Sci. (2021) 22, 4126–4140. doi: 10.3390/ijms22084126, PMID: 33923565 PMC8072586

[32] Falconieri A De Vincentiis S Cappello V Convertino D Das R Ghignoli S. Axonal plasticity in response to active forces generated through magnetic nano-pulling. Cell Rep. (2023) 42:111912. doi: 10.1016/j.celrep.2022.111912, PMID: 36640304 PMC9902337

[33] Falconieri A Folino P Da Palmata L Raffa V. Nano-pulling stimulates axon regeneration in dorsal root ganglia by inducing stabilization of axonal microtubules and activation of local translation. Front Mol Neurosci. (2024) 17:17. doi: 10.3389/fnmol.2024.1340958, PMID: 38633213 PMC11022966

[34] De Vincentiis S Baggiani M Merighi F Cappello V Lopane J Di Caprio M. Low forces push the maturation of neural precursors into neurons. Small. (2023) 19:e2205871–e2205889. doi: 10.1002/smll.202205871 37058009

[35] Cioni JM Lin JQ Holtermann AV Koppers M Jakobs MAH Azizi A. Late endosomes act as mRNA translation platforms and sustain mitochondria in axons. Cell. (2019) 176:56–72.e15. doi: 10.1016/j.cell.2018.11.030, PMID: 30612743 PMC6333918

[36] Zempel H. Genetic and sporadic forms of tauopathies—TAUas a disease driver for the majority of patients but the minority of tauopathies. Cytoskeleton. (2024) 81:66–70. doi: 10.1002/cm.21793, PMID: 37795931

[37] Rogowski K Hached K Crozet C van der Laan S. Tubulin modifying enzymes as target for the treatment of tau-related diseases. Pharmacol Ther. (2021) 218:107681. doi: 10.1016/j.pharmthera.2020.107681, PMID: 32961263

[38] Wang C Ward ME Chen R Liu K Tracy TE Chen X. Scalable production of iPSC-derived human neurons to identify tau-lowering compounds by high-content screening. Stem Cell Reports. (2017) 9:1221–33. doi: 10.1016/j.stemcr.2017.08.019, PMID: 28966121 PMC5639430

[39] Riggio C Calatayud MP Giannaccini M Sanz B Torres TE Fernández-Pacheco R. The orientation of the neuronal growth process can be directed via magnetic nanoparticles under an applied magnetic field. Nanomedicine. (2014) 10:1549–58. doi: 10.1016/j.nano.2013.12.008, PMID: 24407149

[40] Usenovic M Niroomand S Drolet RE Yao L Gaspar RC Hatcher NG. Internalized tau oligomers cause neurodegeneration by inducing accumulation of pathogenic tau in human neurons derived from induced pluripotent stem cells. J Neurosci. (2015) 35:14234–50. doi: 10.1523/JNEUROSCI.1523-15.2015, PMID: 26490863 PMC6605424

[41] Caneus J Akanda N Rumsey JW Guo X Jackson M Long CJ. A human induced pluripotent stem cell-derived cortical neuron human-on-a chip system to study Aβ42 and tau-induced pathophysiological effects on long-term potentiation. Alzheimers Dement. (2020) 6, e12029–e12043. doi: 10.1002/trc2.12029, PMID: 32490141 PMC7253154

[42] Esselun C Dieter F Sus N Frank J Eckert GP. Walnut oil reduces Aβ levels and increases neurite length in a cellular model of early Alzheimer disease. Nutrients. (2022) 14, 1694–1712. doi: 10.3390/nu14091694, PMID: 35565661 PMC9099939

[43] Witte H Neukirchen D Bradke F. Microtubule stabilization specifies initial neuronal polarization. J Cell Biol. (2008) 180:619–32. doi: 10.1083/jcb.200707042, PMID: 18268107 PMC2234250

[44] Li L Yang XJ. Tubulin acetylation: responsible enzymes, biological functions and human diseases. Cell Mol Life Sci. (2015) 72:4237–55. doi: 10.1007/s00018-015-2000-5, PMID: 26227334 PMC11113413

[45] Janke C Magiera MM. The tubulin code and its role in controlling microtubule properties and functions. Nat Rev Mol Cell Biol. (2020) 21:307–26. doi: 10.1038/s41580-020-0214-3, PMID: 32107477

[46] Ballatore C Brunden KR Huryn DM Trojanowski JQ Lee VMY Smith AB. Microtubule stabilizing agents as potential treatment for Alzheimer’s disease and related neurodegenerative Tauopathies. J Med Chem. (2012) 55:8979–96. doi: 10.1021/jm301079z, PMID: 23020671 PMC3493881

[47] Brunden KR Lee VMY Smith AB Trojanowski JQ Ballatore C. Altered microtubule dynamics in neurodegenerative disease: therapeutic potential of microtubule-stabilizing drugs. Neurobiol Dis. (2017) 105:328–35. doi: 10.1016/j.nbd.2016.12.021, PMID: 28012891 PMC5481496

[48] Boiarska Z Passarella D. Microtubule-targeting agents and neurodegeneration. Drug Discov Today. (2021) 26:604–15. doi: 10.1016/j.drudis.2020.11.033 33279455

[49] Michaelis ML Ranciat N Chen Y Bechtel M Ragan R Hepperle M. Protection against beta-amyloid toxicity in primary neurons by paclitaxel (Taxol). J Neurochem. (1998) 70:1623–7. doi: 10.1046/j.1471-4159.1998.70041623.x, PMID: 9523579

[50] Shemesh OA Spira ME. Rescue of neurons from undergoing hallmark tau-induced Alzheimer’s disease cell pathologies by the antimitotic drug paclitaxel. Neurobiol Dis. (2011) 43:163–75. doi: 10.1016/j.nbd.2011.03.008, PMID: 21406229

[51] Zhang B Maiti A Shively S Lakhani F McDonald-Jones G Bruce J. Microtubule-binding drugs offset tau sequestration by stabilizing microtubules and reversing fast axonal transport deficits in a tauopathy model. Proc Natl Acad Sci. (2005) 102:227–31. doi: 10.1073/pnas.0406361102, PMID: 15615853 PMC544048

[52] Brunden KR Zhang B Carroll J Yao Y Potuzak JS Hogan AML. Epothilone D improves microtubule density, axonal integrity, and cognition in a transgenic mouse model of Tauopathy. J Neurosci. (2010) 30:13861–6. doi: 10.1523/JNEUROSCI.3059-10.2010, PMID: 20943926 PMC2958430

[53] Zhang B Carroll J Trojanowski JQ Yao Y Iba M Potuzak JS. The microtubule-stabilizing agent, Epothilone D, reduces axonal dysfunction, neurotoxicity, cognitive deficits, and Alzheimer-like pathology in an interventional study with aged tau transgenic mice. J Neurosci. (2012) 32:3601–11. doi: 10.1523/JNEUROSCI.4922-11.2012, PMID: 22423084 PMC3321513

[54] Barten DM Fanara P Andorfer C Hoque N Wong PYA Husted KH. Hyperdynamic microtubules, cognitive deficits, and pathology are improved in tau transgenic mice with low doses of the microtubule-stabilizing agent BMS-241027. J Neurosci. (2012) 32:7137–45. doi: 10.1523/JNEUROSCI.0188-12.2012, PMID: 22623658 PMC6622320

[55] Penazzi L Tackenberg C Ghori A Golovyashkina N Niewidok B Selle K. Aβ-mediated spine changes in the hippocampus are microtubule-dependent and can be reversed by a subnanomolar concentration of the microtubule-stabilizing agent epothilone D. Neuropharmacology. (2016) 105:84–95. doi: 10.1016/j.neuropharm.2016.01.002, PMID: 26772969 PMC4873443

[56] Makani V Zhang B Han H Yao Y Lassalas P Lou K. Evaluation of the brain-penetrant microtubule-stabilizing agent, dictyostatin, in the PS19 tau transgenic mouse model of tauopathy. Acta Neuropathol Commun. (2016) 4:106. doi: 10.1186/s40478-016-0378-4, PMID: 27687527 PMC5043530

[57] Zhang B Yao Y Cornec AS Oukoloff K James MJ Koivula P. A brain-penetrant triazolopyrimidine enhances microtubule-stability, reduces axonal dysfunction and decreases tau pathology in a mouse tauopathy model. Mol Neurodegener. (2018) 13:59. doi: 10.1186/s13024-018-0291-3, PMID: 30404654 PMC6223064

[58] Guo B Huang Y Gao Q Zhou Q. Stabilization of microtubules improves cognitive functions and axonal transport of mitochondria in Alzheimer’s disease model mice. Neurobiol Aging. (2020) 96:223–32. doi: 10.1016/j.neurobiolaging.2020.09.011, PMID: 33039900

[59] Cross DJ Huber BR Silverman MA Cline MM Gill TB Cross CG. Intranasal paclitaxel alters Alzheimer’s disease phenotypic features in 3xTg-AD mice. J Alzheimers Dis. (2021) 83:379–94. doi: 10.3233/JAD-210109, PMID: 34308901

[60] Yao Y Muench M Alle T Zhang B Lucero B Perez-Tremble R. A small-molecule microtubule-stabilizing agent safely reduces Aβ plaque and tau pathology in transgenic mouse models of Alzheimer’s disease. Alzheimers Dement. (2024) 20:4540–58. doi: 10.1002/alz.13875, PMID: 38884283 PMC11247666

[61] Karahalil B Yardım-Akaydin S Baytas SN. An overview of microtubule targeting agents for cancer therapy. Arhiv za Higijenu Rada i Toksikologiju Sciendo. (2019) 70:160–72. doi: 10.2478/aiht-2019-70-3258, PMID: 32597128

[62] Cooper JM Lathuiliere A Migliorini M Arai AL Wani MM Dujardin S. Regulation of tau internalization, degradation, and seeding by LRP1 reveals multiple pathways for tau catabolism. J Biol Chem. (2021) 296:100715. doi: 10.1016/j.jbc.2021.100715, PMID: 33930462 PMC8164048

[63] Shi JM Zhu L Lan X Zhao DW He YJ Sun ZQ. Endocytosis is a key mode of interaction between extracellular β-amyloid and the cell membrane. Biophys J. (2020) 119:1078–90. doi: 10.1016/j.bpj.2020.07.035, PMID: 32857960 PMC7499104

[64] Li S Hong S Shepardson NE Walsh DM Shankar GM Selkoe D. Soluble oligomers of amyloid β protein facilitate hippocampal long-term depression by disrupting neuronal glutamate uptake. Neuron. (2009) 62:788–801. doi: 10.1016/j.neuron.2009.05.012, PMID: 19555648 PMC2702854

[65] Taniguchi K Yamamoto F Amano A Tamaoka A Sanjo N Yokota T. Amyloid-β oligomers interact with NMDA receptors containing GluN2B subunits and metabotropic glutamate receptor 1 in primary cortical neurons: relevance to the synapse pathology of Alzheimer’s disease. Neurosci Res. (2022) 180:90–8. doi: 10.1016/j.neures.2022.03.001, PMID: 35257837

[66] Al Kabbani MA Wied T Adam D Klimek J Zempel H. Knockdown of TTLL1 reduces Aβ-induced TAU pathology in human iPSC-derived cortical neurons (2024). 10.3390/pharmaceutics18081038 PMC13516559 42654155

[67] Buchholz SKMBSMZH. The tau isoform 1N4R confers vulnerability of MAPT knockout human iPSC-derived neurons to amyloid beta and phosphorylated tau-induced neuronal dysfunction. Alzheimers Dement. (2025):e14403. doi: 10.1002/alz.14403, PMID: 40019008 PMC12089071

[68] Bullmann T Holzer M Mori H Arendt T. Pattern of tau isoforms expression during development *in vivo*. Int J Dev Neurosci. (2009) 27:591–7. doi: 10.1016/j.ijdevneu.2009.06.001, PMID: 19540327

[69] Raffa V. Force: a messenger of axon outgrowth. Semin Cell Dev Biol. (2022) 140:3–12. doi: 10.1016/j.semcdb.2022.07.004, PMID: 35817654

[70] De Vincentiis S Merighi F Blümler P De La Ossa Guerra JG Di Caprio M Onorati M. 3D-printed weight holders design and testing in mouse models of spinal cord injury. Front Drug Deliv. (2024) 4:4. doi: 10.3389/fddev.2024.1397056, PMID: 40260418

[71] Pardridge WM. Blood-brain barrier drug targeting: the future of brain drug development. Mol Interv. (2003) 3:90–105. doi: 10.1124/mi.3.2.90, PMID: 14993430

[72] Huang Y Zhang B Xie S Yang B Xu Q Tan J. Superparamagnetic Iron oxide nanoparticles modified with tween 80 pass through the intact blood-brain barrier in rats under magnetic field. ACS Appl Mater Interfaces. (2016) 8:11336–41. doi: 10.1021/acsami.6b02838, PMID: 27092793

[73] Qiao R Jia Q Hüwel S Xia R Liu T Gao F. Receptor-mediated delivery of magnetic nanoparticles across the blood-brain barrier. ACS Nano. (2012) 6:3304–10. doi: 10.1021/nn300240p, PMID: 22443607

