# Supplementary figures and images for: Mechanical stimulation prevents impairment of axon growth and overcompensates microtubule destabilization in cellular models of Alzheimer’s disease and related Tau pathologies
Source: Front Med (Lausanne). 2025 May 14;12:1519628. doi: 10.3389/fmed.2025.1519628 (PMC12117335; doi:10.3389/fmed.2025.1519628)

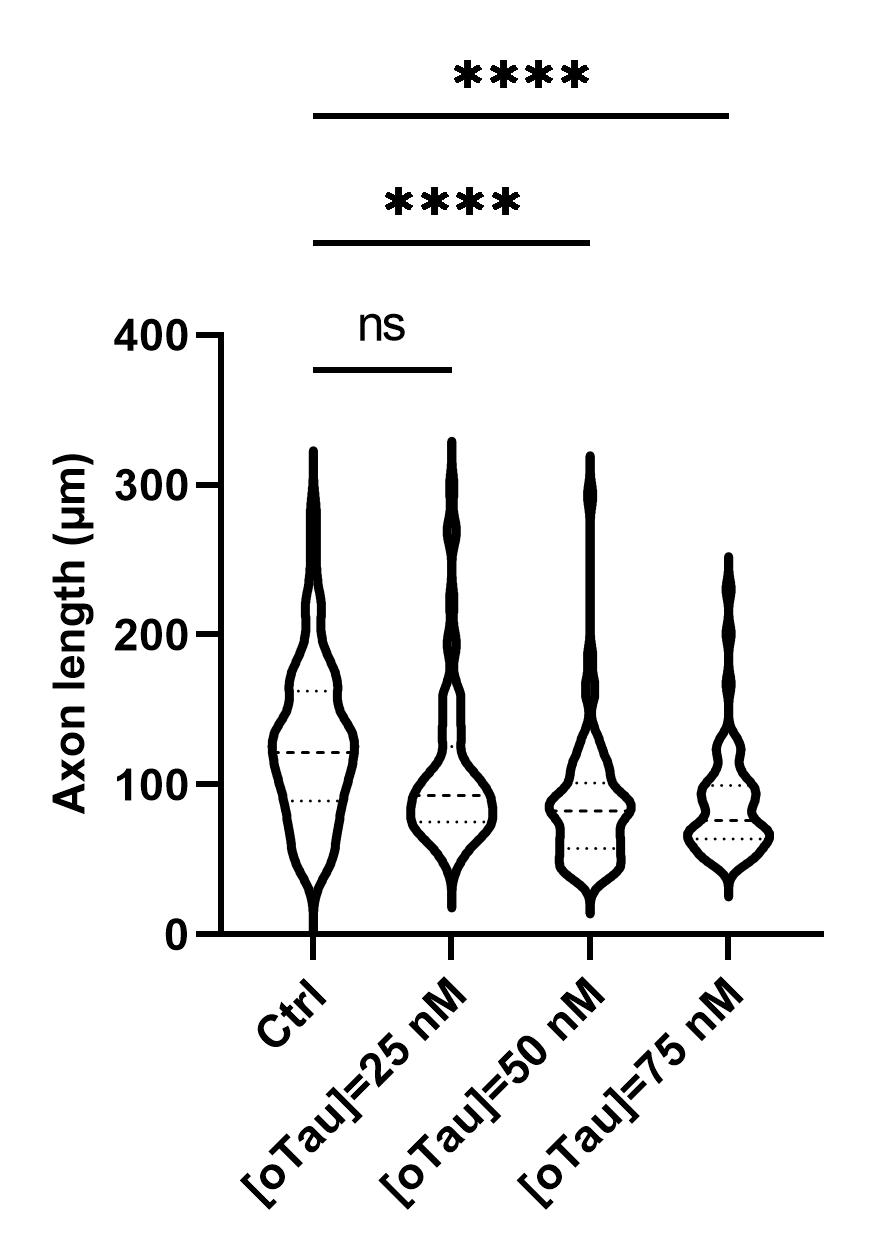

Supplement: SUPPLEMENTARY FIGURE S1 — Effects of different concentrations of oTau on primary mouse HNs. Elongation analysis of primary mouse HNs treated with [Tau] = 25 nM, [Tau] = 50 nM, and [Tau]=75 nM versus control condition. Violin plot, data are expressed as median (dashed line) and 25–75 percentiles (dotted lines). n = 60, from two replicates. Kruskal-Wallis test for unpaired data followed by Dunn’s multiple comparisons test, two-tailed. Mean rank of each group is compared with mean rank of control group. **** = p < 0.0001. A shortening of axons in the treated samples compared to control samples was observed. oTau concentration of 50 nM was chosen, as the lower concentration (25 nM) had no statistically significant effect compared to control neurons, and the higher concentration (75 nM) showed no dose-dependent effect compared to 50 nM. [file Image_1.JPEG]

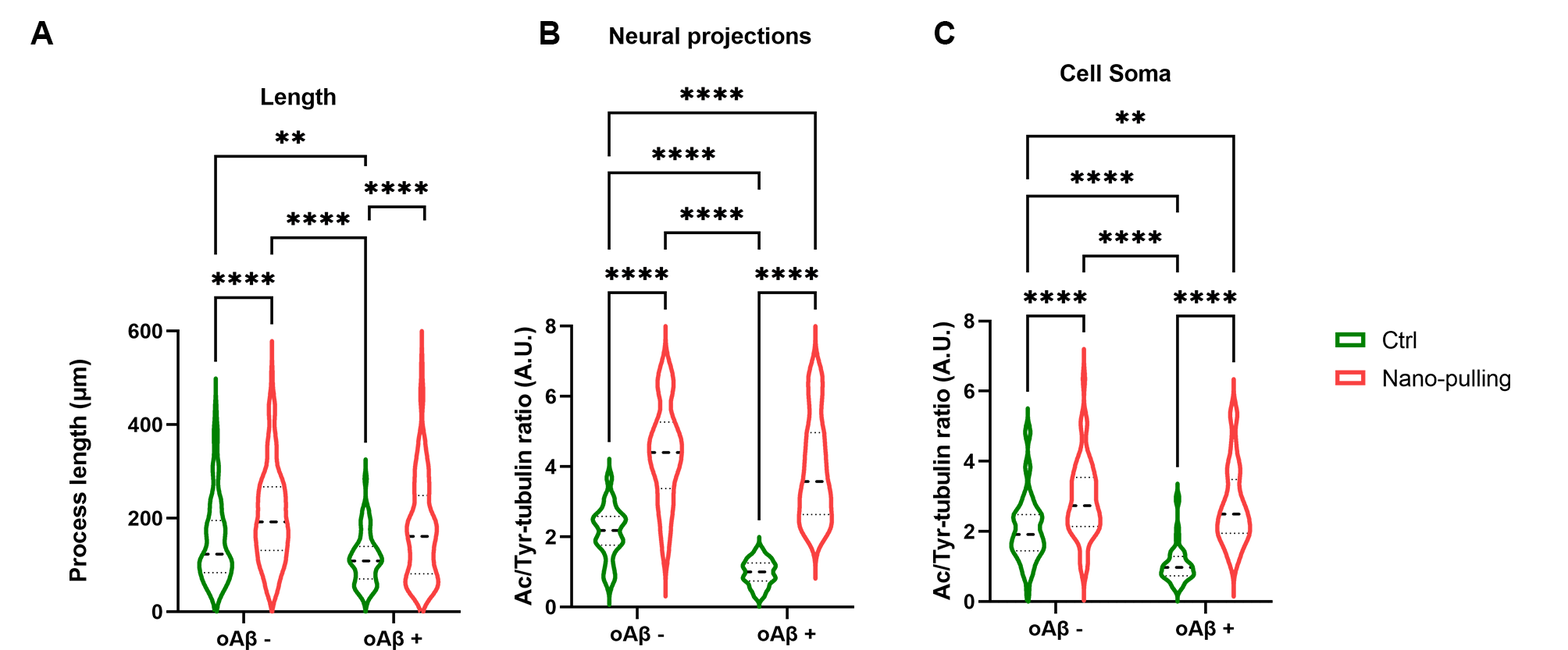

Supplement: SUPPLEMENTARY FIGURE S2 — Effects of oTau and nano-pulling on iNs—all controls. (A) Elongation analysis of hiPSC-derived cortical iNs treated with 1 μM oAβ and subjected to nano-pulling. Violin plot, data are expressed as median (dashed line) and 25–75 percentiles (dotted lines). n = 120, from four replicates. Kruskal-Wallis test for unpaired data followed by Dunn’s multiple comparisons test, two-tailed. Interaction: F (1, 476) = 0.3023, p = 0.58. Row Factor (oligomer treatment): F (1, 476) = 16.98, p < 0.0001. Column Factor (nano-pulling): F (1, 476) = 47.44, p < 0.0001. (B) Analysis of MT stability in neural processes of iNs treated with 1 μM oAβ and “stretched” with mechanical stimulation. Violin plot, data are expressed as median (dashed line) and 25–75 percentiles (dotted lines). n = 48 from four replicates. Kruskal-Wallis test for unpaired data followed by Dunn’s multiple comparisons test, two-tailed. Interaction: F (1, 188) = 5.381, p = 0.02. Row Factor (oligomer treatment): F (1, 188) = 25.42, p < 0.0001. Column Factor (nano-pulling): F (1, 188) = 275, p < 0.0001. (C) Somatic MT stability analysis of iNs treated with 1 μM oAβ and “stretched” with mechanical stimulation. Violin plot, data are expressed as median (dashed line) and 25–75 percentiles (dotted lines). n = 50 from four replicates. Kruskal-Wallis test for unpaired data followed by Dunn’s multiple comparisons test, two-tailed. Interaction: F (1, 202) = 8.963, p = 0.031. Row Factor (oligomer treatment): F (1, 202) = 19.80, p < 0.0001. Column Factor (nano-pulling): F (1, 202) = 89.08, p < 0.0001. [file Image_2.TIF]
